# Soft Coral-Derived Lemnalol Alleviates Monosodium Urate-Induced Gouty Arthritis in Rats by Inhibiting Leukocyte Infiltration and iNOS, COX-2 and c-Fos Protein Expression

**DOI:** 10.3390/md11010099

**Published:** 2013-01-10

**Authors:** Hsin-Pai Lee, Shi-Ying Huang, Yen-You Lin, Hui-Min Wang, Yen-Hsuan Jean, Shu-Fen Wu, Chang-Yih Duh, Zhi-Hong Wen

**Affiliations:** 1 Department of Marine Biotechnology and Resources, Asia-Pacific Ocean Research Center, National Sun Yat-sen University, Kaohsiung 80424, Taiwan; E-Mails: hplee0929@gmail.com (H.-P.L.); johnjohnkings@gmail.com (S.-Y.H.); chas6119@gmail.com (Y.-Y.L.); yihduh@mail.nsysu.edu.tw (C.-Y.D.); 2 Department of Orthopaedic Surgery, Ping-Tung Christian Hospital, 60, Ta-Lian Road, Ping-Tung 90059, Taiwan; E-Mail: jean.tang@msa.hinet.net; 3 Department of Fragrance and Cosmetic Science, Center of Excellence for Environmental Medicine, Kaohsiung Medical University, Kaohsiung 80708, Taiwan; E-Mail: davidw@kmu.edu.tw; 4 Department of Life Science, Institute of Molecular Biology, National Chung-Cheng University, Chia-Yi 62102, Taiwan; E-Mail: biosfw@ccu.edu.tw

**Keywords:** gout, lemnalol, monosodium urate, allodynia, inflammation

## Abstract

An acute gout attack manifests in the joint as dramatic inflammation. To date, the clinical use of medicinal agents has typically led to undesirable side effects. Numerous efforts have failed to create an effective and safe agent for the treatment of gout. Lemnalol—an extract from Formosan soft coral—has documented anti-inflammatory and anti-nociceptive properties. In the present study, we attempt to examine the therapeutic effects of lemnalol on intra-articular monosodium urate (MSU)-induced gouty arthritis in rats. In the present study, we found that treatment with lemnalol (intramuscular [im]), but not colchicine (oral [po]), significantly attenuated MUS-induced mechanical allodynia, paw edema and knee swelling. Histomorphometric and immunohistochemistry analysis revealed that MSU-induced inflammatory cell infiltration, as well as the elevated expression of c-Fos and pro-inflammatory proteins (inducible nitric oxide synthase and cyclooxygenase-2) observed in synovial tissue, were significantly inhibited by treatment with lemnalol. We conclude that lemnalol may be a promising candidate for the development of a new treatment for gout and other acute neutrophil-driven inflammatory diseases.

## 1. Introduction

The acute gout attack is a dramatic inflammatory response, often appearing in the joint. The inflammatory process is initiated by the deposition of monosodium urate (MSU) crystals in the surrounding tissue [1,2]. Therapeutic options for the management of acute gout include nonsteroidal anti-inflammatory drugs (NSAIDs), colchicine (low dose), glucocorticoids and intra-articular steroids. NSAIDs can also induce adverse gastrointestinal ulceration or bleeding and renal dysfunction. The use of colchicine can also induce diarrhea. The adverse effects of steroid use include hyperglycemia, fluid retention and mood alteration. A small, uncontrolled pilot study also reported that interleukin (IL)-1β inhibitors improved acute gout symptoms [1]. 

Clinically, gout is associated with edema and erythema of the joints, as well as severe pain. Several studies have indicated that the intra-articular (ia) injection of MSU elicited symptoms similar to those observed in clinical gout [2,3,4]. The ia MSU induced joint swelling, limping and nociceptive behaviors, including mechanical allodynia and thermal hyperalgesia, and Intense infiltration of neutrophils into the joint space was also observed [2,3,5]. Neutrophils accumulate in both the joint fluid and the synovial membrane, generating pro-inflammatory mediators that trigger membranolysis [6]. It has been know that MSU can upregulate the expression of proinflammatory proteins, inducible nitric oxide synthase (iNOS) and cyclooxygenase-2 (COX-2) expression in monocytes/macrophages [7,8]. COX-2 and iNOS are thought to be responsible for prostaglandin and nitric oxide expression, respectively, observed in gouty arthritis [8]. Previous studies have reported the upregulation of c-Fos protein in the synovium and chondrocytes of arthritic patients. Trabandt *et al.* (1992) found that the activation of c-Fos signaling plays an important role in arthritic joint destruction [9]. In the previous study, a rat model of MSU-induced joint arthritis was used to evaluate the efficacy of various analgesic and anti-arthritic agents [2,10].

The marine organisms that inhabit soft coral produce a diverse group of biologically active substances, including polypeptides and terpenoids [11]. These substances exhibit cytotoxic, anti-inflammatory and antimicrobial properties [12]. Soft corals have been recognized as a rich source of sesquiterpenoids, which exhibit cytotoxic, anti-inflammatory and antimicrobial properties [12]. Lemnalol (8-isopropyl-5-methyl-4-methylene-decahydro-1,5-cyclo-naphthalen-3-ol) is a ylangene-type sesquiterpenoid extracted from Japanese soft coral (*Lemnalia tenuis*), as well as Verseveldt and Fosmasan soft corals (*Lemnalia cervicorni*) [13,14]. Our previous study had found that lemnalol significantly inhibits the expression of iNOS and COX-2 in lipopolysaccharide (LPS)-stimulated macrophages, with significant antinociceptive effects in carrageenan-induced inflammation and chronic constriction injury-induced neuropathy in rats [13,15]. On the basis of these results, we propose that lemnalol may help in the treatment of gouty arthritis.

In the present study, we intended to evaluate the effect of lemnalol on painful-response testing and histopathological changes, as well as elucidate the anti-inflammatory mechanisms underlying these effects of lemnalol in MSU-challenged rats. As a positive control for these experiments, we used colchicine, which can decrease leukocyte migration and phagocytosis and be effective in controlling short-term episodes of human gouty arthritis [4]. 

## 2. Results

### 2.1. The Effects of Lemnalol on the Inflammation Induced by MSU

**Figure 1:**
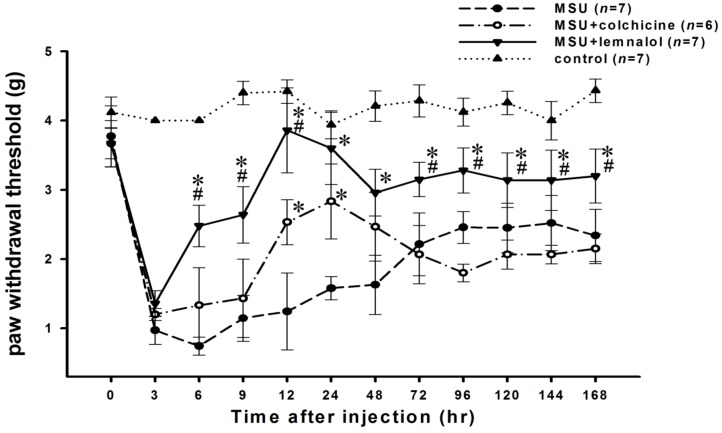
The effect of lemnalol on monosodium urate (MSU)-induced nociception. Paw-withdrawal thresholds to ankle pressure with von Frey hairs on ankles for ia MSU-injected rats, either untreated or treated with therapeutic agents. Each rat in the MSU experimental group received an ia MSU injection in the ankle. Each animal in the control group received an ia injection of saline in the ankle.A time course showing the effect of systemic lemnalol 30 mg/kg im or colchicine 1.5 mg/kg (po) on MSU-induced mechanical allodynia in ankle joints. The paw-withdrawal threshold decreased progressively, reaching a maximum value from 3 to 9 h after MSU injection. Lemnalol significantly attenuated MSU-induced mechanical allodynia to a greater extent than achieved with colchicine (*p < 0.05 as compared to the MSU group; #*p* < 0.05 as compared to the MSU + colchicine group).

The ia injection of MSU crystals into the ankle joints of rats induced a significant decrease in the paw-withdrawal threshold. The mechanical allodynia behavior, captured as paw-withdrawal threshold, decreased progressively with time, reaching a maximum value from 3 to 9 h after MSU injection and then increasing gradually 48–72 h after injection. The co-administration of a 30 mg/kg intramuscular (im) dose of lemnalol significantly inhibited the MSU-induced decrease in paw-withdrawal threshold during the period ranging from 6 to 168 h. Oral colchicine (1.5 mg/kg) significantly attenuated MSU-induced nociception from 12 to 24 h. The anti-nociceptive activity of lemnalol was more potent than that of colchicine (Figure 1).

**Figure 2:**
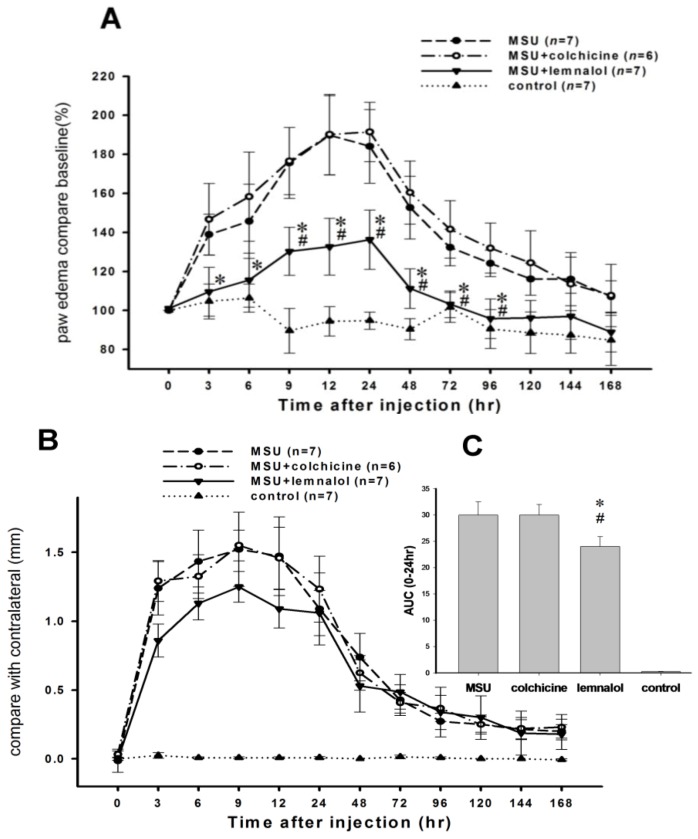
The effect of lemnalol on MSU-induced ankle edema and knee swelling. Each rat in the MSU experimental group received an ia MSU injection in the knee or ankle, while each animal in the control group received an ia injection of saline in the knee or ankle. (**A**) A time course showing the effect of systemic lemnalol 30 mg/kg (im) and colchicine 1.5 mg/kg (po) on MSU-induced edema in ankle joints. Lemnalol, but not colchicine, significantly inhibited MSU-induced paw edema; (**B**) A time course showing the effect of systemic lemnalol and colchicine on MSU-induced swelling in the knee joint; (**C**) The area under the effect-time curve for knee swelling in the first 24 h after MSU injection showed that the lemnalol group had significantly less knee swelling as compared with the colchicine group (*p < 0.05 compared to the MSU group; #*p* < 0.05 compared to the MSU + colchicine group).

Paw edema increased significantly from 3 to 72 h after the ia injection of MSU into the ankle joint. Paw edema improved in the MSU + lemnalol group as compared with the MSU-ankle alone group. Colchicine (1.5 mg/kg) attenuated nociception, but had no significant effect on paw edema after the MSU injection (Figure 2A). As shown in Figure 2B, ia injections of MSU into knee joints induce a significant increase in the width of the knee joint. There were no significant differences among the MSU, MSU + lemnalol and MSU + colchicine groups. The results were calculated as AUCs (from 0 to 12 h after the MSU injection) to simplify comparisons between groups. Lemnalol was more effective than colchicine in attenuating MSU-induced knee joint swelling (Figure 2C).

### 2.2. Histomorphometric Analysis of the Effect of Lemnalol on MSU-Induced Infiltration

**Figure 3:**
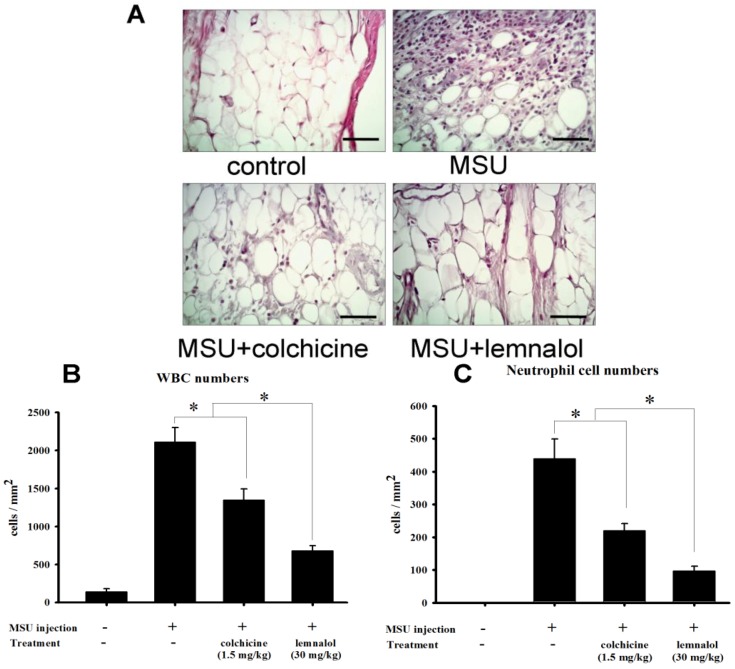
Histomorphometric analysis of the effect of lemnalol on MSU-induced infiltration. (**A**) Representative photographs of the synovial tissue from control, MSU, MSU + colchicine and MSU + lemnalol groups after staining with hematoxylin and eosin. 24 h after the injection of MSU crystals, histologic analysis of infrapatellar synovium and subsynovial tissue demonstrated a marked increase in the number of neutrophils. Lemnalol and colchicine reduced the severity of the MSU crystal-induced neutrophil infiltration. There was a marked reduction in neutrophil influx in the lemnalol group; (**B**) WBC (white blood cell) count in the ankle-joint synovium (WBC cell per square millimeter) (**C**) Neutrophil count in the ankle-joint synovium (neutrophils per square millimeter). WBC and neutrophil counts were both elevated in the MSU-ankle group as compared to the control-ankle group. The MSU-ankle + colchicine group exhibited significantly reduced WBC and neutrophil counts compared with the MSU-ankle group. The MSU-ankle + lemnalol group exhibited significantly lower WBC and neutrophil counts compared with the MSU-ankle + colchicine group. Scale bars: 50 μm for all images (*p < 0.05 compared to the MSU group; #*p* < 0.05 compared to the MSU + colchicine group).

Synovial specimens were obtained from the inflamed ankle joints in rats, 24 h after injection with MSU crystals. These specimens were then stained with hematoxylin and eosin. The ia injections of MSU elicited pronounced leukocyte infiltration (primarily neutrophils) of the superficial synovium neutrophils (Figure 3A). Co-treatment with colchicine or lemnalol inhibited leukocyte infiltration (Figure 3A). As shown in Figure 3B,C, the number of WBC (white blood cell) and neutrophils was significantly increased in the control and MSU groups. In the MSU + colchicine group as compared with the MSU group, the number of WBCs and neutrophils decreased significantly. The MSU + lemnalol group exhibited decreased levels of WBCs and neutrophils as compared with the MSU + colchicine group (Figure 3B,C).

### 2.3. Effects of Lemnalol on iNOS, COX-2 and c-Fos Protein Expression in Ankle Synovium

**Figure 4:**
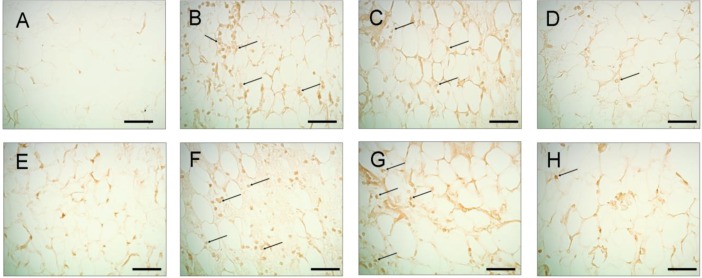
The effects of lemnalol on synovial iNOS and COX-2 protein expression in rats with MSU-induced arthritis. iNOS staining of synovial sections at 24 h after normal saline injection (**A**); MSU injection (**B**); MSU + colchicine injection (**C**); and MSU + lemnalol injection (**D**); MSU-induced synovitis was associated with upregulated iNOS protein expression. There were markedly fewer immunopositive cells in the lemnalol-treated group. The upregulation of iNOS-immunoreactive cells was inhibited in the lemnalol-treated group. Synovial sections obtained at 24 h after normal saline injection (**E**); MSU injection (**F**); MSU + colchicine injection (**G**); and MSU + lemnalol injection (**H**) and subsequently stained for COX-2. MSU-induced synovitis was associated with increased COX-2 protein expression. There were markedly fewer inflammatory cells in the lemnalol-treated group. The upregulation of COX-2-immunoreactive cells was inhibited in the lemnalol-treated group. Scale bars: 50 μm for all images.

**Figure 5:**
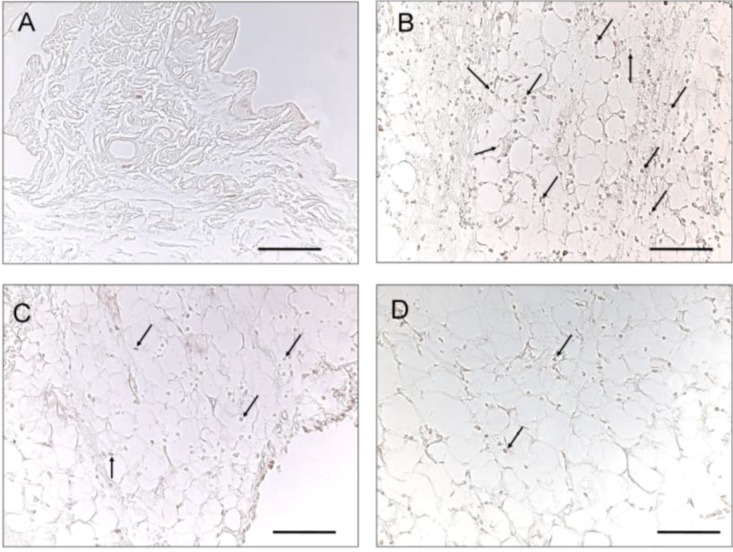
The effect of lemnalol on synovial c-Fos protein expression in a rat model of MSU-induced arthritis.c-Fos staining in synovial sections obtained at 24 h after injection with normal saline (**A**); MSU (**B**); MSU + colchicine (**C**); and MSU + lemnalol (**D**). Intramuscular lemnalol inhibited the MSU-induced upregulation of c-Fos immunoreactivity in synovial tissue. Colchicine exhibited weak inhibition of the MSU-induced upregulation of c-Fos immunoreactivity. Scale bars: 100 μm for all images.

Figure 4 illustrates the levels of ankle synovium iNOS and COX-2 immunoreactivity in control (Figure 4A,E), MSU (Figure 4B,F), MSU + colchicine (Figure 4C,G) and MSU + lemnalol rats (Figure 5D,H). Fewer iNOS- and/or COX-2-expressing cells were observed in the synovium of control rats. 24 h after the administration of MSU injections to the ankle joint, numerous iNOS- and/or COX-2-immunoreactive cells were observed in the ankle joint synovium. The co-administration of lemnalol markedly reduced the increase in iNOS- (Figure 4D) and COX-2-immunoreactive (Figure 4H) cells in the ankle-joint synovium of MSU-injected rats as compared to those injected with MSU + colchicine. Figure 5 shows the level of c-Fos immunoreactivity in ankle synovium in control (Figure 5A), MSU (Figure 5B), MSU + colchicine (Figure 5C) and MSU + lemnalol rats (Figure 5D). No c-Fos immunoreactivity was observed in the control group. The marked upregulation of immunoreactive cells was apparent in synovial tissue from the MSU group. Both MSU + colchicine and MSU + lemnalol reduced the number of c-Fos-immunoreactive cells. Lemnalol attenuated MSU-induced c-Fos immunoreactivity. Colchicine only weakly inhibited the MSU-induced upregulation of c-Fos immunoreactivity (Figure 5C).

## 3. Discussion

### 3.1. Lemnalol Attenuates MSU-Induced Inflammation

The articular injection of MSU is thought to be painful for rats and rabbits. In such animal models, the injection causes joint swelling and elicits limping, mechanical allodynia and thermal hyperalgesia; further, foot-withdrawal thresholds decrease after MSU injection [2,3]. The injection of MSU to induce joint arthritis has been studied in rats, rabbits, cats and dogs [2,16,17,18]. Several aspects of the inflammatory response were altered, including neutrophil accumulation and joint size. Arthritis scores increased and cytokine levels subsided by 48–72 h after MSU injection. Previous studies reported neutrophil influx to joint fluid from 16 to 24 h after MSU injection (ia), as well as intense infiltration of the superficial and subsynovial lining layer of the synovium by leukocytes (primarily neutrophils) at 12 h and 24 h [3,4]. MSU induced the infiltration of macrophage and mast cells, which release mediators, including IL-1β and tryptase, which are in turn involved in MSU-induced pain [19,20,21,22]. Similarly, the present study found that ia MSU significantly increased the infiltration of WBC and neutrophils in ankle tissue. This increase in WBC and neutrophil infiltration was accompanied by inflammation, nociception and swelling. The intramuscular injection of lemnalol significantly reduced the severity of mechanical allodynia and ankle/knee swelling. Lemnalol also significantly attenuated the MSU-induced infiltration of neutrophils and WBC. Notably, colchicine did not diminish ankle and knee joint swelling in MSU-induced arthritis. In comparison to lemnalol, colchicine weakly inhibited WBC and neutrophil infiltration.

### 3.2. Effects of Lemnalol on iNOS and COX-2 in MSU-Induced Gouty Arthritis

The clinical hallmarks of joint inflammation include severe pain, edema and erythema. These effects are mediated by prostanoids, such as PGE_2_. *In vitro* studies have shown that MSU stimulates COX-2 protein expression in human monocytes and triggers the production of PGE_2_ [23]. Kant *et al.* demonstrated that hyperuricemia results in the upregulation of COX-2 immunoreactivity in kidney tissue [24]. Moreover, intra-articular MSU-induced synovial COX-2 gene expression [25]. This evidence suggests that the upregulation of COX-2 expression plays an important role in the inflammation associated with ia MSU-induced arthritis. 

Nitric oxide mediates the body’s innate immune response. Extracellular stimuli can activate NO signaling pathways to initiate iNOS expression [26]. Nitric oxide also mediates inflammation in the context of arthritis [27]; mononuclear cells from rheumatoid arthritis patients exhibited higher iNOS expression and activity [28]. Similarly, synovial tissue from gouty arthritis patients exhibits elevated iNOS expression. iNOS protein and mRNA expression were also upregulated in response to *in vitro* MSU stimulation of monocytes/macrophages [7]. The results of our study showed that lemnalol, but not colchicine, significantly attenuated the MSU-induced upregulation of iNOS and COX-2 expression in synovial tissue.

### 3.3. The Effect of Lemnalol on c-Fos in MSU-Induced Gouty Arthritis

The AP-1 (activator protein 1) transcription factor is a dimeric complex that comprises members of the JUN, FOS and ATF (activating transcription factor) families [29]. c-Fos protein expression is upregulated in the synovial tissue of rheumatoid arthritis and osteoarthritis patients [30,31,32]. Increased c-fos gene expression triggers the synthesis and secretion of matrix metalloproteinases, which destroy arthritic joints [31,32,33,34]. Moreover, c-fos/c-jun heterodimers play important roles in regulating the expression of IL-1β, IL-6, TNF-α and collagenase [35,36,37]. However, to date, the role of c-Fos in gouty arthritis is unclear. Figure 5 depicts the marked upregulation of c-FOX immunoreactivity observed in MSU synovial tissue. To our knowledge, this is the first study to report ia MSU-induced c-Fos protein expression in synovium. Lemnalol significantly attenuated MSU-induced c-Fosregulation. We propose that lemnalol might inhibit joint destruction by regulating synovial c-Fos expression.

### 3.4. Colchicine and Lemnalol Dosages in the Present Study

To calculate the human-equivalent doses based on body weight, we divided the rat dose by 6.2 [38]. Colchicine doses from 0.5 to 0.8 mg/kg may induce bone marrow failure and carry a 10% chance of mortality in humans; the ingestion of >0.8 mg/kg is fatal [39]. In rats, a dose of 2 mg/kg carries a 75% chance of mortality [40]. In the present study, we selected 1.5 mg/kg as the maximal dose of colchicine. Colchicine exhibited only limited effects in terms of relieving pain, decreasing swelling and inhibiting neutrophil infiltration and protein expression. As reported previously [13], lemnalol (15 mg/kg) treatment achieved a slight, but not significant inhibition of the nociceptive response in ia MSU-challenged rats. We therefore selected a lemnalol dose of 30 mg/kg for our experiments. The results showed that the anti-inflammatory effects of lemnalol were more potent than that of colchicine. As compared with lemnalol, colchicine exhibited only a weak capacity to reduce ankle and knee swelling and block neutrophil infiltration. Lemnalol was more effective than colchicine in inhibiting the upregulation of iNOS and COX-2 protein expression triggered by MSU-induced gouty arthritis. Furthermore, we found that colchicine (1.5 mg/kg), but not lemnalol (30 mg/kg), induced loose stool in rats (data not shown). Treatment with lemnalol alone did not cause any obvious external behavior side effects, including diarrhea and locomotor dysfunction, which represents another advantage over colchicine.

### 3.5. Summary

The present study shows that intra-articular MSU-induced nociception, neutrophil infiltration and the expression of pro-inflammatory proteins iNOS and COX-2, as well as transcription factor c-Fos in ankles. Both the nociceptive and swelling effects of MSU were inhibited by lemnalol. The anti-inflammatory effects of lemnalol were also observed in rats with MSU-induced gouty arthritis in which lemnalol suppressed MSU-induced inflammatory cell infiltration, as well as iNOS, COX-2 and c-Fos protein expression in ankle synovial tissue. These inflammatory effects of MSU-induced gouty arthritis were inhibited by lemnalol. 

## 4. Material and Methods

### 4.1. Animals

The male Wistar rats (250–300 g) used for the experiment were obtained from LASCO Inc., Taipei, Taiwan. The rats were housed in a Plexiglass cage and monitored daily under a controlled temperature (22 °C) with a 12-h light/dark cycle and freely available food and water. The use of rats conformed to the Guiding Principles in the Care and Use of Animals as approved by the Council of the American Physiology Society and approved by the National Sun Yat-Sen University Animal Care and Use Committee. All possible efforts were made to minimize animal suffering and reduce the number of animals used. Each rat was used only once during this study. 

### 4.2. MSU Preparation and the Gout Animal Model

The method for crystal preparation proposed by Coderre *et al.*, 1987 [2], and Getting *et al.*, 2002, with slight modifications, was used for this study [4]. Briefly, 1 g of uric acid (catalog No. 1198772, Sigma, St. Louis, MO, USA) was dissolved in 200 mL of boiling water containing 6 mL of 1 N NaOH. The pH value of the final solution was adjusted to 7.2 through the addition of HCl. The solution was cooled and stirred at room temperature, then stored overnight at 5 °C. The precipitate was filtered from the solution, dried under low heat and sifted through a 250-pm wire mesh. The urate crystals were then mixed in an injection solution of 10% Tween 80 in 0.9% saline. Gout was induced with an ia injection of the prepared MSU (1 mg/0.1 mL) into the right knee or ankle joint, after each animal was anesthetized with 2.5% isoflurane in an air/O_2_ mixture.

### 4.3. Experimental Design

Rats were allocated randomly to 8 groups: a control-knee group (*n* = 7), control-ankle group (*n* = 7), MSU-knee group (*n* = 7), MSU-ankle group (*n* = 7), MSU-knee + colchicine group (*n* = 6), MSU-ankle + colchicine group (*n* = 6), MSU-knee + lemnalol group (*n* = 7) and MSU-ankle + lemnalol group (*n* = 7). Each rat in the MSU experimental groups received an ia MSU injection in the knee or ankle, while each animal in the control group received an ia injection of 0.1 mL saline in the knee or ankle. Immediately after administration of the MSU injection, each rat in the MSU experimental group received colchicine 1.5 mg/kg (po) and lemnalol 30 mg/kg (im). The behavior of each rat included in the study was observed from 0 h (baseline, before the ia injection) to 168 h. The animals were then sacrificed. For the statistical analysis, the area under the curve (AUC) in the plot of paw edema (0–12 h) *versus* time was calculated using the trapezoidal method [41].

### 4.4. Measurement of Nociceptive Behavior (Mechanical Allodynia)

In order to assess mechanical allodynia, the hindpaw withdrawal thresholds (PWT; in g) were measured using calibrated von Frey filaments (North Coast Medical, Inc., Morgan Hill, CA, USA). The diameters of the filaments corresponded to a logarithmic scale of the force exerted, which allowed the perceived intensity to be measured on a linear and interval scale. The rat cages were placed in a rack on an elevated metal mesh floor, which permitted easy access to their paws. A series of von Frey filaments of logarithmically incremental stiffness (0.2–10.0 g) were applied to the mid-plantar region of the hindpaw. Chaplan’s “up-down” method, which involves the use of alternate large and small fibers, was used to determine the 50% withdrawal threshold [42]. Each filament was placed in contact with the paw surface for 5 s, 5 times at approximately 3-min intervals. Any sign of discomfort (vocalization, flinching) or attempt of the animal to withdraw the paw was considered as a positive response [15,42]. 

### 4.5. Measurement of Knee Width and Ankle Edema

Knee width was measured to determine the amount of tissue swelling as an index of inflammation. Rats were anesthetized briefly with 2% isoflurane and then the width of the knee joint was measured using calipers (series No. 560, Mitytoyo, Kanagawa, Japan) after the injection of MSU. After the injection of MSU, paw volume was measured using a paw volume meter (plethysmometer, Singa Inc., Taipei, Taiwan). The change in paw volume was calculated by subtracting the initial paw volume (basal) from the paw volume measured at each time-point [13].

### 4.6. Histopathological Examination

Rats were sacrificed by inducing deep anesthesia with 2% isoflurane at 168 h after the induction of gout. Each rat was then perfused intracardially with 400 mL of ice-cold phosphate-buffered saline containing 1% sodium nitrite and heparin (0.2 U/mL), followed by freshly prepared 4% paraformaldehyde in 0.1 M phosphate-buffered saline (pH 7.4). The knee- and ankle-joint samples were sectioned 0.5 cm above and below the joint line, fixed in 10% neutral buffered formalin for 3 days and then decalcified for 2 weeks in buffered 12.5% ethylenediaminetetraacetic acid (EDTA) with 10% neutral buffered formalin. The specimens were dehydrated in a graded series of alcohol (Tissue-Tek, Sakura Finetek Japan Co., Ltd., Tokyo, Japan) and embedded in paraffin. Next, 2-μm sections (HM340E, Microm, Biotechnical Services, Inc., San Diego, CA, USA) were prepared for hematoxylin and eosin staining. Additional 1-μm sections were prepared for immunohistochemistry to assess morphological joint changes. Each section was examined under an upright microscope (DM 6000B, Leica Inc., Wetzlar, Germany) and digital-image output system (idea SPOT, Diagnostic instruments Inc., Sterling Heights, MI, USA). Histopathological examination was used to determine the number of infiltrating cells and to characterize the neutrophil and WBC populations present [43,44,45].

### 4.7. Immunohistochemistry for iNOS, COX-2 and c-Fos

The joint samples were processed for immunochemical staining as described in previous studies [13,15]. Each 1-μm section from a paraffin-embedded sample was mounted on a slide, deparaffinized with xylene and dehydrated using a graded ethanol series. Next, endogenous peroxidase was quenched with 3% H_2_O_2_ for 8 min. The antigen was retrieved by enzymatic digestion with proteinase K (20 mM; Sigma, St Louis, MO, USA) in PBS for 45 min and then washed with Tris-buffered saline. Non-specific adsorption was minimized by incubating the sections in 5% normal horse serum in PBS for 30 min. The sections were incubated overnight at 4 °C with anti-iNOS (1:300 dilution; BD Pharmingen, San Diego, CA, USA; catalog No. 6103322; polyclonal antibody), anti-COX-2 (1:300, Cayman Chemical, Ann Arbor, MI, USA; catalog No. 160106; polyclonal antibody) and anti-c-Fos (1:300 dilution; Calbiochem, La Jolla, CA, USA) antibodies. Specific labeling was detected with a biotin-conjugated anti-rabbit IgG (Vector Laboratories, Burlingame, CA, USA) and avidin-biotin peroxidase in combination with an ABC kit (Vectastain ABC kit; Vector Labs, Burlingame, CA, USA). Finally, the sections were reacted with 3,3′-diaminobenzidine tetrahydrochloride (DAB) for 1–2 min. All slides for immunochemical staining were analyzed using an upright microscope (DM 6000B, Leica Inc., Wetzlar, Germany) in combination with a digital image output system (idea SPOT, Diagnostic instruments Inc., Sterling Heights, MI, USA).

### 4.8. Statistical Analysis

All data are presented as the mean ± standard error of the mean (SEM). The AUCs for the time-response curves were calculated for individual animals using sigmaPlot software, with time on the X-axis and response on the Y-axis. For statistical analysis, all of the data were analyzed using a one-way analysis of variance, followed by the Student-Newman-Keuls post-hoc test for multiple comparisons. A significant difference was defined as *p* < 0.05.

## 5. Conclusions

In the present study, lemnalol exerted anti-inflammatory and analgesic effects that included the inhibition of neutrophil infiltration and the attenuation of knee and ankle joint swelling in MSU-induced gouty arthritis. Markedly fewer inflammatory cells were observed in the lemnalol-treated group. Immunohistochemistry studies demonstrated that lemnalol inhibited upregulation of iNOS, COX-2 and c-Fos protein expression triggered by the MSU challenge. We conclude that lemnalol represents a potential candidate for the treatment of gout and other acute neutrophil-driven inflammatory disease. It may also have therapeutic potential for patients with inflammatory arthritis.
